# Supplementary figures and images for: Comparative RNA-Seq analyses of Drosophila plasmatocytes reveal gene specific signatures in response to clean injury and septic injury
Source: PLoS One. 2020 Jun 29;15(6):e0235294. doi: 10.1371/journal.pone.0235294 (PMC7323993; doi:10.1371/journal.pone.0235294)

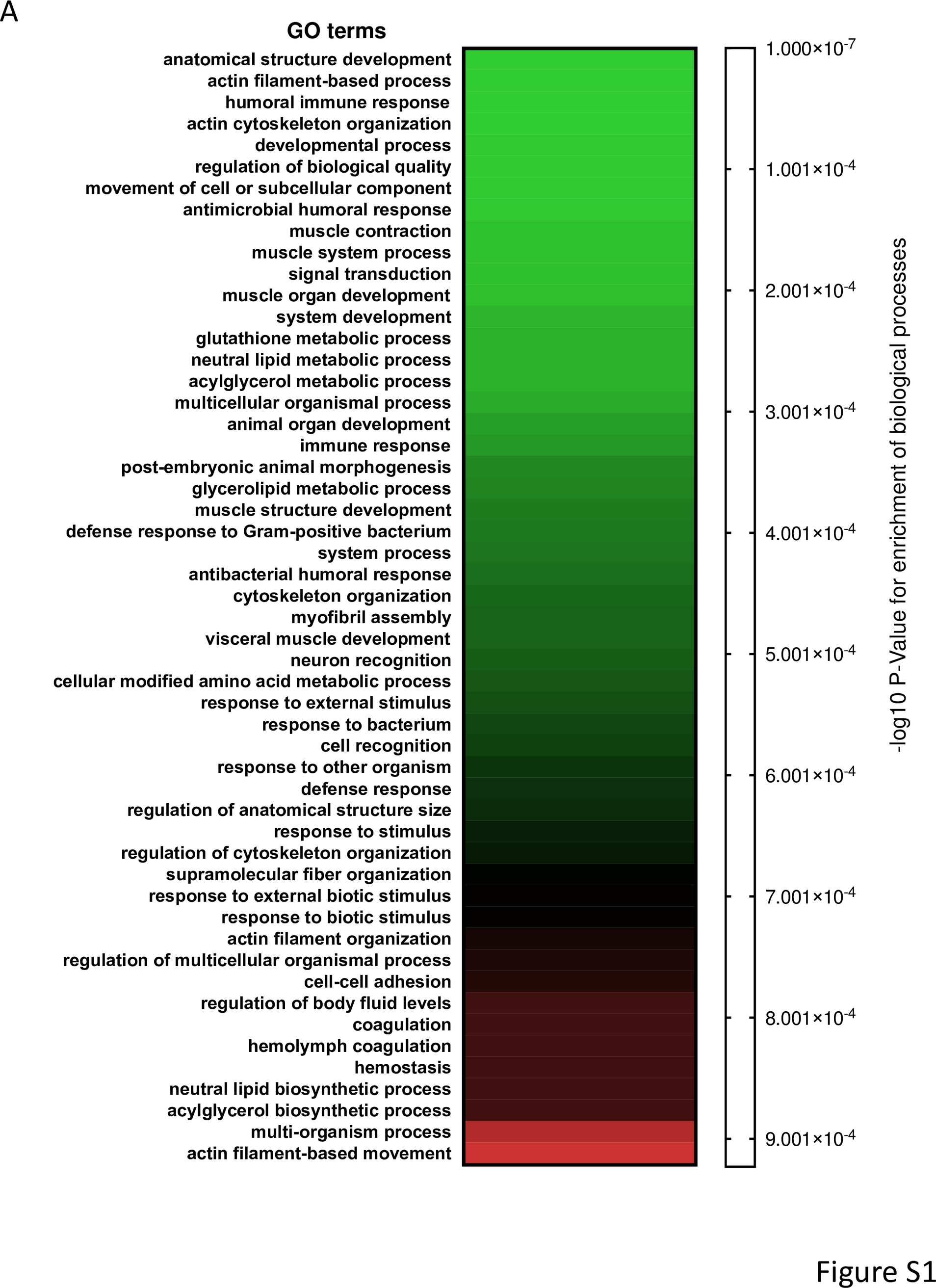

Supplement: S1 Fig — (TIF) [file pone.0235294.s001.tif]

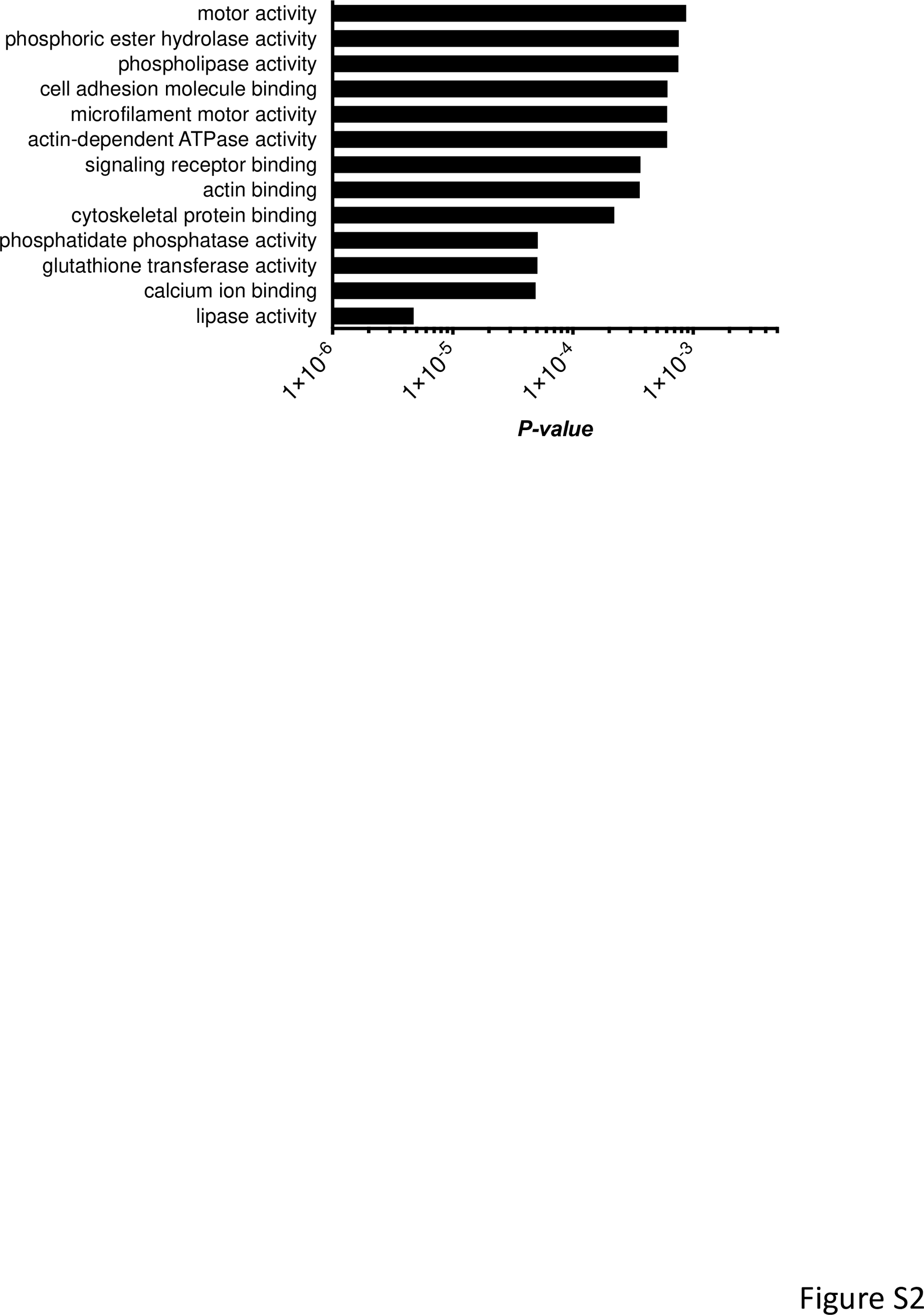

Supplement: S2 Fig — (TIF) [file pone.0235294.s002.tif]
