# Supplementary material for: Comparative RNA-Seq analyses of Drosophila plasmatocytes reveal gene specific signatures in response to clean injury and septic injury
Source: PLoS One. 2020 Jun 29;15(6):e0235294. doi: 10.1371/journal.pone.0235294 (PMC7323993; doi:10.1371/journal.pone.0235294)
Supplement: S1 Table — (DOCX) [file pone.0235294.s003.docx]

Table S1

| CG | Full name | FC |  | CG | Full name | FC |
| --- | --- | --- | --- | --- | --- | --- |
| Extracellular matrix | | |  | Membrane trafficking | | |
| Extracellular matrix components | | |  | Rab GTPases family | | |
| *CG6953* | *fat-spondin* | 19.10 |  | *CG5771* | *Rab11* | 6.35 |
| *CG9280* | *Glutactin* | 18.62 |  | *CG3870* | *RabX1* | 4.93 |
| *CG6378* | *Secreted protein, acidic, cysteine-rich* | 16.22 |  | *CG9575* | *Rab35* | 3.98 |
| *CG2198* | *Amalgam* | 9.40 |  | *CG6601* | *Rab6* | 2.97 |
| *CG3322* | *Laminin B2* | 8.74 |  | *CG17060* | *Rab10* | 2.66 |
| *CG7123* | *Laminin B1* | 7.21 |  | *CG8287* | *Rab8* | 2.37 |
| *CG16858* | *viking* | 6.40 |  | *CG3269* | *Rab2* | 2.19 |
| *CG10236* | *Laminin A* | 5.78 |  | *CG4921* | *Rab4* | 2.02 |
| *CG3083* | *Peroxiredoxin 6005* | 6.62 |  | Vesicle trafficking | | |
| *CG5826* | *Peroxiredoxin* | 3.14 |  | *CG4764* | *Vacuolar protein sorting 29* | 9.21 |
| Cytoskeleton organization | | |  | *CG10711* | *Vacuolar protein sorting 36* | 4.46 |
| GTPases superfamily | | |  | *CG4071* | *Vacuolar protein sorting 20* | 4.14 |
| *CG9366* | *Rho-like* | 5.97 |  | *CG14804* | *Vacuolar protein sorting 26* | 4.04 |
| *CG12530* | *Cdc42* | 4.44 |  | *CG6259* | *Vacuolar protein sorting 60* | 3.43 |
| *CG8416* | *Rho1* | 4.24 |  | *CG14750* | *Vacuolar protein sorting 25* | 3.06 |
| *CG8556* | *Rac2* | 4.18 |  | *CG17828* | *Vacuolar protein sorting 37A* | 2.71 |
| *CG2248* | *Rac1* | 2.89 |  | *CG12770* | *Vacuolar protein sorting 28* | 2.13 |
| Actin binding proteins | | |  | *CG5625* | *Vacuolar protein sorting 35* | 2.13 |
| *CG8936* | *Arp2/3 complex, subunit 3B* | 27.62 |  | *CG7146* | *Vacuolar protein sorting 39* | 2.01 |
| *CG8978* | *Arp2/3 complex , subunit 1* | 11.92 |  |  |  |  |
| *CG32858* | *singed* | 9.91 |  |  |  |  |
| *CG30173* | *Haematopoietic stem/progenitor cell protein 300* | 7.39 |  |  |  |  |
| *CG5869* | *Glia maturation factor* | 6.54 |  |  |  |  |
| *CG12363* | *Dynein light chain 90F* | 6.51 |  |  | | |
| *CG9881* | *Arp2/3 complex , subunit 5* | 6.43 |  |  |  |  |
| *CG15112* | *enabled* | 6.33 |  |  |  |  |
| *CG9553* | *Chickadee* | 5.80 |  |  |  |  |
| *CG4636* | *SCAR* | 4.74 |  |  |  |  |
| *CG10954* | *Arp2/3 complex , subunit 2* | 4.74 |  |  |  |  |
| *CG9115* | *myotubularin* | 4.27 |  |  |  |  |
| *CG3265* | *Eb1* | 4.14 |  |  |  |  |
| *CG4931* | *specifically Rac1-associated protein 1* | 3.90 |  |  |  |  |
| *CG7558* | *Actin-related protein 3* | 3.88 |  |  |  |  |
| *CG9749* | *Abelson interacting protein* | 3.72 |  |  |  |  |
| *CG9901* | *Actin-related protein 2* | 2.82 |  |  |  |  |
| *CG4560* | *Arp2/3 complex , subunit 3A* | 2.05 |  |  |  |  |
